# Quercetin-loaded exosomes derived from human umbilical cord mesenchymal stem cells alleviate microglia-mediated neuroinflammation via an anti-inflammatory mechanism

**DOI:** 10.1007/s10787-026-02236-z

**Published:** 2026-04-21

**Authors:** Sajeda Osman, Gulen Melike Demirbolat, Sevim Isik

**Affiliations:** 1 Department of Molecular Biology, Institute of Science, Uskudar University, Uskudar, Istanbul 34662 Turkey; 2 Department of Pharmaceutical Technology, Faculty of Pharmacy, Acibadem Mehmet Ali Aydinlar University, Istanbul, Turkey; 3 Stem Cell Research and Application Center (USKOKMER), Uskudar University, Uskudar, Istanbul 34662 Turkey; 4 Department of Molecular Biology and Genetics, Faculty of Engineering and Natural Sciences, Uskudar University, Uskudar, Istanbul 34662 Turkey

**Keywords:** Neuroinflammation, Lipopolysaccharide, Microglia, Mesenchymal stem cells, Exosomes, Quercetin

## Abstract

Neuroinflammation is central to the progression of neurodegenerative diseases (NDs), with microglia contributing through the release of pro-inflammatory and oxidative mediators. Although quercetin (Que) possesses potent anti-inflammatory and antioxidant properties, its clinical application is limited by poor solubility and bioavailability. Umbilical cord mesenchymal stem cells (UC-MSCs)-derived exosomes (Exo) offer a promising delivery system to enhance Que efficacy. This study investigated the anti-inflammatory effects of Que-loaded Exo (Que-Exo) using an in vitro neuroinflammation model. Exo were isolated from UC-MSC-conditioned media via ultracentrifugation and loaded with Que through sonication. Characterization of Exo and Que-Exo was performed using dynamic light scattering, zeta potential analysis, transmission electron microscopy, and western blotting. In vitro release and cellular uptake studies assessed Que-Exo release profiles and their internalization by HMC3 microglial cells. Neuroinflammation was induced in HMC3 cells using lipopolysaccharide (LPS). The effects of Que-Exo on pro-inflammatory cytokine and enzyme expression, nitric oxide (NO) production, and inflammatory signaling pathways were evaluated using flow cytometry, the Griess assay, and western blotting. In vitro release from Que-Exo demonstrated improved stability and controlled delivery, while fluorescence imaging confirmed efficient uptake of Que-Exo by HMC3 cells. Mechanistically, Que-Exo inhibited NF-κB nuclear translocation, a key regulator of pro-inflammatory mediator transcription. Que-Exo pretreatment significantly reduced TNF-α, IL-6, iNOS, and COX-2 expression and suppressed NO synthesis in LPS-stimulated cells. Overall, Que-Exo exhibited superior anti-inflammatory activity compared to Exo and free Que, highlighting the synergistic effect of exosomal delivery in enhancing Que bioavailability and therapeutic efficacy against neuroinflammation.

**Graphical Abstract:**

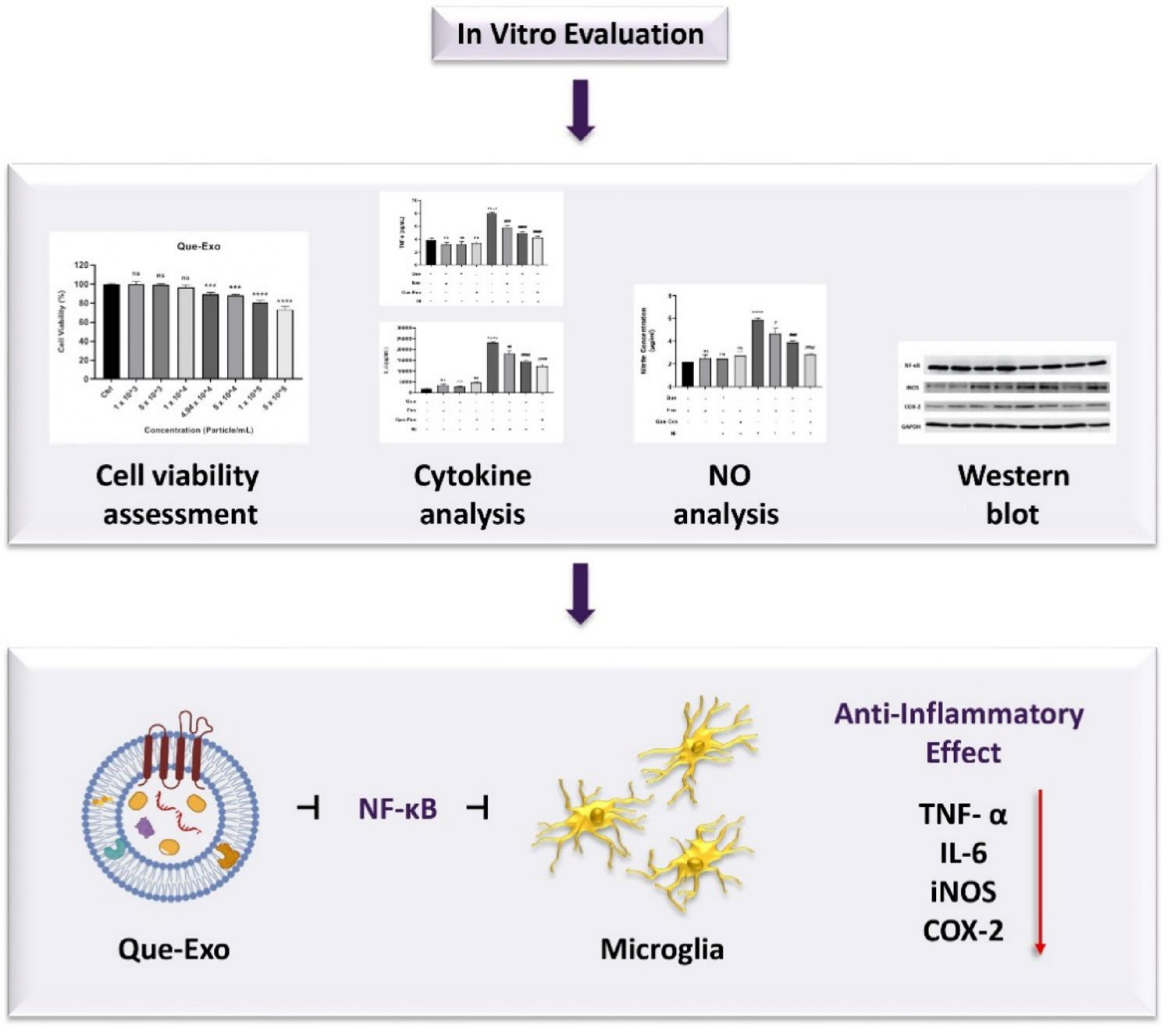

## Introduction

Microglia are the primary immune cells residing within the central nervous system (CNS) and play a vital role in maintaining brain health, responding to injury, and modulating neural networks, as described by Colonna and Butovsky (2017) and Peña-Ortega (2015). These specialized cells continuously monitor the brain environment and respond to various stimuli, both pathological and physiological. Their ability to detect and respond to changes allows them to function as the first line of defense against potential threats, such as pathogens and tissue damage (de Araújo Boleti et al. 2020). Streit et al. (2004) and Zhang et al. (2023) demonstrated that microglia are key cellular mediators of neuroinflammatory processes and are essential for maintaining tissue homeostasis, removing invading pathogens, and promoting recovery from injuries. Additionally, they contribute to neurodegenerative diseases (NDs) and brain inflammation, as reported by Harry and Kraft (2012) and Shabab et al. (2017). Neuroinflammation and neurotoxicity are associated with the proinflammatory state of microglia, which is often triggered by factors such as pathogens, tissue damage, neurotoxins, infections, or injury. Neuroinflammation can be acute or chronic, and it is essential to understand both in the context of CNS pathology. Acute inflammation is a rapid response involved in tissue repair, typically triggered by injury or infection (Isik et al. 2023). Conversely, according to Streit et al. (2004) and Yeman-Kıyak et al. (2025), chronic inflammation is linked to NDs, leading to neuronal dysfunction, damage, and ultimately disease progression. Krasemann et al. (2017) and Sadeghdoust et al. (2024) illustrated that chronic microglial activation induces the expression of multiple genes and proteins, including inducible nitric oxide synthase (iNOS) and pro-inflammatory cytokines such as interleukin-1 beta (IL-1β), tumor necrosis factor-alpha (TNF-α), cyclooxygenase-1 (COX-1), cyclooxygenase-2 (COX-2), and reactive oxygen species (ROS). In chronic neuroinflammation, microglia remain activated for extended periods, continuously releasing neurotoxic chemicals and cytokines that contribute to long-term neurodegeneration.

For example, systemic inflammatory response syndrome is triggered by lipopolysaccharide (LPS), an endotoxin found in the outer membrane of gram-negative bacteria, via Toll-like receptor (TLR) signalling. When LPS binds to TLR4 on the surface of microglia, it triggers multiple signal transduction pathways, such as the mammalian target of rapamycin (mTOR), mitogen-activated protein kinase (MAPK), and phosphoinositide 3-kinase/protein kinase B (PI3K/AKT). Ultimately, these pathways result in the activation of NF-κB. The subsequent generation of pro-inflammatory cytokines, chemokines, and inducible enzymes, such as COX-2 and iNOS, is mediated by the activation of NF-κB, which results in neuroinflammation (Shabab et al. 2017). Neuroinflammatory responses have various effects on the immune system, physiology, metabolism, and psychology of the body. Furthermore, the length and progression of the initial stimulation determine the extent of neuroinflammation (DiSabato et al. 2016).

Owing to the pivotal role of microglia in neuroinflammation, their dysregulation has become a significant therapeutic target in NDs, leading to increased interest in stem cell-based approaches to regulate microglial activity and facilitate neuronal regeneration (Shen et al. 2017). Abbaszadeh et al. (2020) and Harrell et al. (2021) indicated that exosomes, nano-sized extracellular vesicles secreted by various cell types, have emerged as promising alternatives to address several limitations of stem cell therapy. These vesicles promote intercellular communication and delivering proteins, lipids, and nucleic acids to mitigate neuroinflammation and enhance neural repair, as clarified by He et al. (2023) and Krylova and Feng (2023). According to Abbaszadeh et al. (2020) and He et al. (2023), umbilical cord mesenchymal stem cells (UC-MSCs)-derived exosomes (Exo) are particularly advantageous among various stem cells because of their low immunogenicity, ethical accessibility, and potent anti-inflammatory and neuroprotective properties. These vesicles mimic the therapeutic effects of their parental MSCs but offer advantages, including improved safety, lower immunogenicity, and the ability to cross the blood–brain barrier (BBB), making them a promising cell-free delivery system for NDs.

To augment the therapeutic potential of Exo, some bioactive compounds with recognized anti-inflammatory properties have been explored for their synergistic effects on neuroinflammatory pathways (Isik et al. 2025). Quercetin (Que; 3,3′,4′,5,7-pentahydroxyflavone), a flavonoid found in vegetables, fruits, and herbs, is known for its antioxidant, anti-inflammatory, and neuroprotective properties (Jazvinšćak Jembrek et al. 2021). Previous studies have shown that Que can inhibit the activation of nuclear factor kappa-light-chain-enhancer of activated B cells (NF-κB), ultimately decreasing the expression of the pro-inflammatory cytokines TNF-α, IL-1β, and Interleukin-6 (IL-6) (Cui et al. 2022), and the enzymes iNOS and COX-2. It also promotes the activation of nuclear factor erythroid 2-related factor 2 (Nrf2), a key regulator of cellular antioxidant responses, and inhibits the activation of the NOD-like receptor pyrin domain-containing protein 3 (NLRP3) inflammasome, thereby contributing to the attenuation of neuroinflammation, as explained by Jazvinšćak Jembrek et al. (2021) and Han et al. (2021). Despite these benefits, the clinical application of Que is limited by its low water solubility, instability, and poor bioavailability (Gonçalves et al. 2015).

In this study, we aimed to investigate whether Que-loaded Exo (Que-Exo) derived from UC-MSCs exert anti-inflammatory effects in an LPS-induced neuroinflammation model using the human microglial HMC3 cell line. Additionally, we explored the molecular mechanisms underlying Que-Exo activity by analyzing their impact on the NF-κB signaling pathway and their role in modulating pro-inflammatory cytokines and enzymes.

## Materials and methods

### Culture of primary UC-MSCs

UC-MSCs were obtained from The American Type Culture Collection (ATCC, PCS-500–010) and cultured in DMEM-LG (Dulbecco’s modified eagle medium–low glucose, Gibco, 11,885,084) supplemented with 10% FBS (Fetal bovine serum, Cytiva SH30071.03 M) and 1% penicillin–streptomycin (Capricon Scientific, PS-B). Cells were maintained at 37 °C in a humidified atmosphere containing 5% CO₂. Subculturing was performed when cell confluency reached approximately 80%, and cells were seeded at a density of 1 × 10^4^ cells/cm^2^.

To minimize FBS-derived Exo contamination, FBS was processed using Amicon® Ultra-Centrifugal Filters; 100 kDa (Sigma-Aldrich, UFC900324). FBS was centrifuged at 3,000×g for 90 min at 4°C, and the resulting ultrafiltrate was collected and stored at –20°C. For the preparation of Exo-depleted medium, 10% of the ultrafiltrate was added to DMEM-LG containing 1% penicillin/streptomycin. After 48 h of incubation, the conditioned medium from UC-MSCs at passage 5 was collected for subsequent Exo isolation.

### Culture of HMC3 microglial cell line

Human microglial clone 3 cell line (HMC3) were obtained from The American Type Culture Collection (ATCC, CRL-3304) and cultured in MEM (Minimum essential medium, Capricorn Scientific, MEM-XA) supplemented with 1% L-glutamine (Capricorn Scientific, GLN.B), 1% sodium pyruvate (Capricorn Scientific, NPY-B), 10% FBS (Gibco, 10,270,106), and 1% penicillin–streptomycin. The medium was renewed daily, and the cells were maintained at 37 °C in a humidified incubator with 5% CO₂. Subculturing was performed when cell confluency reached approximately 80%, and they were seeded at a density of 2 × 10^4^ cells/cm^2^.

To induce a neuroinflammation (NI) model, the cells at 70–80% confluency were first stimulated with 50 μM ATP (Adenosine triphosphate, Thermo Fisher Scientific, R0441) for 10 min. This was followed by treatment with 1 μg/mL of LPS (Sigma-Aldrich, L4391-1MG) and 10 ng/mL of IFN-γ (Interferon gamma, Gibco, PHC4031), then the cells were incubated under these conditions for 48 h. HMC3 cells were pretreated with Que-Exo for 1 h, followed by stimulation with ATP, LPS, and IFN-γ for 48 h to induce NI.

### Isolation of UC-MSCs-derived Exo

Exo were isolated from UC-MSCs using an ultracentrifuge (Beckman Coulter Optima Max-XP). The culture media were first centrifuged at 300 × g for 10 min to remove dead cells, and the supernatant was centrifuged again at 10,000 × g for 30 min to remove cell debris. The supernatant was passed through a 0.22 μm filter and centrifuged in ultracentrifuge tubes at 100,000 × g for 70 min. The Exo pellets were resuspended in PBS (Phosphate-buffered saline, Sigma-Aldrich, P4417) and centrifuged again at 100,000 × g for 70 min at 4 °C. Finally, the pellet containing Exo was suspended in PBS and stored at − 80 °C (Théry et al. 2006).

### Preparation of Que-Exo

Que (MedChemExpress, HY-18095) was loaded into Exo using sonication. A stock solution was prepared by mixing 1 mg of Que in a vial with 4 mL of Exo sample and placed under a sonicator (Bandelin Sonopuls Ultrasonic Homogenizer). The following settings were used with 20% amplitude: 6 cycles of ultrasonication were applied for 3 s on/off for 3 min with a 2-min cooling period in an ice-water bath between each cycle. After sonication, the prepared Que-Exo were incubated at 37 °C for 1 h to allow recovery of the exosomal membrane. Que-Exo were separated by centrifugation at 10,000xg for 10 min at 4 °C. Que in Exo was analyzed using UV–VIS spectrophotometer (Thermo Fisher Scientific). Before sample analysis, a stock Que solution in methanol was used, with maximum absorption peaks observed at 256 and 370 nm. Because of the higher consistency at 256 nm, absorbance measurements at this wavelength were used for all calculations (Demirbolat et al. 2022). The absorbance of the supernatant was recorded before and after the loading process. A set of Que solutions in methanol at different concentrations was analyzed, and a graph of absorbance versus known concentration was drawn to obtain the calibration curve.

### Characterization of Exo and Que-Exo and labeling of Que-Exo

The concentration and size distribution of Exo and Que-Exo were analyzed using dynamic light scattering (DLS) with a Wyatt DynaPro NanoStar device. Zeta potential measurements, derived from electrophoretic mobility, were assessed using an Anton Paar Litesizer 500 to evaluate the colloidal stability. For morphological analysis, samples were imaged using transmission electron microscopy (TEM) with a Thermo Scientific™ Talos L120C. Western blotting was performed to confirm the presence of exosomal surface markers, including CD63 (1:1000, Affinity, AF5117), CD81 (1:1000, Affinity, DF2306), and CD9 (1:1000, Affinity, AF5139).

For cellular uptake analysis, Que-Exo were labeled with Cytotrace^TM^ CM-DiD (AAT Bioquest, 22060) by incubating 300 μL of the sample with 5 μM dye at 37°C for 30 min. Excess dye was removed using Amicon® Ultra-Centrifugal Filters (100 kDa) and washed with 10 mL ddH_2_O, followed by centrifugation at 3,000×g for 30 min at 4°C. Labeled Que-Exo were incubated with HMC3 cells seeded in 24-well plates for 3, 6, and 24 h. After fixation with 4% paraformaldehyde, the nuclei were stained with DAPI (neoFroxx, 1322MG005), and the samples were mounted with ProLong™ Glass Antifade Mountant (Thermo Fisher Scientific, P36980). Fluorescent imaging was performed using an Axio Observer 3 microscope (Carl Zeiss, Oberkochen, Germany). To quantify the extent of internalization, the Mean Fluorescence Intensity (MFI) of the CM-DiD signal was calculated at each time point using ImageJ software. Additionally, cellular uptake was quantified as the percentage of CM-DiD-positive cells relative to the total number of cells.

### Determination of encapsulation efficiency percentage for Que-Exo

The Que content in Exo was determined indirectly by measuring the drug present in the external phase. Unloaded Que was removed by centrifuging the solution at 10,000 × g for 10 min at 4 °C. The supernatant was transferred to a clean Eppendorf tube, and the pellet (unloaded Que, which is poorly water-soluble) was dissolved in methanol to calculate the loading efficiency. The supernatant containing Que-Exo was then analyzed for drug content using a UV–VIS spectrophotometer at 256 nm. The amount of Que was calculated using a previously constructed calibration curve. The percentage of drug encapsulation in Exo was determined using the following equation:

$$\begin{aligned} {\mathrm{Encapsulation}}\,{\mathrm{Efficiency}}\,({\mathrm{EE}})\% & {\text{ = }}\frac{{{\mathrm{Total}}\,{\mathrm{amount}}\,{\mathrm{of}}\,{\mathrm{drug}} - {\mathrm{Amount}}\,{\mathrm{of}}\,{\mathrm{free}}\,{\mathrm{drug}}}}{{{\mathrm{Total}}\,{\mathrm{amount}}\,{\mathrm{of}}\,{\mathrm{drug}}}} \\ & \quad \times {\mathrm{100}} \\ \end{aligned}$$

### In vitro release assessment of Que-Exo

The in vitro release from Que-Exo and Que dispersions was evaluated using the dialysis bag method. A 12–14 kDa pore-size dialysis membrane was pre-soaked in PBS for 1 day to eliminate excess glycerin in the membrane before assembling the Franz Cell system (Phoenix DB-6) to evaluate the release of Que from Exo. The system was maintained at 37 °C with a stirring speed of 200 rpm. The reception chamber of the Franz Cells was filled with 6.5 mL of PBS (pH 7.4) at 37 °C and a magnetic stirring bar was added to the chamber. For the control group, Que was prepared as a physical dispersion in PBS (pH 7.4). To accurately simulate the challenges of free drug delivery, no surfactants or organic stabilizers were used. The mixture was briefly sonicated to ensure a uniform primary distribution before being placed in the release apparatus. The experiment began by adding 1 mL of Que-Exo or 1 mL of Que dispersed in PBS to the donor chambers of the Franz CellsSamples were collected at predetermined time intervals: 10, 20 min, 1, 6, and 24 h. The samples were analyzed using UV–VIS spectrophotometry to generate drug release profiles over time. Data were collected to construct a graph of Que release (%) over time.

### Cell viability assay

A cell viability assay was conducted using the MTT method (Roche, 11,465,007,001) to evaluate the effect of the NI model, Que, Exo, and Que-Exo. HMC3 microglial cells were plated in 96-well plates at a density of 1 × 10^4^ cells/well and treated with various concentrations of the respective agents. Triplicate wells were used for each experiment. After 24 h of treatment, MTT reagent was added to the cells, and after 4 h, a solubilization buffer was added. The cells were incubated at 37 °C with 5% CO_2_ for 16 h. Finally, absorbance measurements were performed at 570 nm using a microplate reader to assess cell viability.

### Pro-inflammatory cytokine analysis by flow cytometry

Cell culture supernatants were collected 48 h after NI induction and stored at − 20 °C until analysis. The levels of the pro-inflammatory cytokines TNF-α and IL-6 secreted by HMC3 microglial cells were quantified using the LEGENDplex™ Human Essential Immune Response Panel (BioLegend, 740,929) according to the manufacturer’s instructions. This bead-based multiplex assay enabled the simultaneous detection of TNF-α and IL-6 cytokines using flow cytometry (Attune™ NxT Acoustic Focusing Cytometer; Thermo Fisher Scientific), providing high sensitivity and specificity.

### Determination of NO scavenging activity

NO production was assessed by quantifying its stable metabolite, nitrite, present in the supernatant using the Griess reagent assay (Cell Signaling Technology, 13,547). The supernatants were collected from each group, and 50 µL was added to a 96-well plate, similar to the standards. Subsequently, 50 µL of Griess Reagent (1:1 ratio of sulfanilamide and NED solutions) was added to each well. Following a 10-min incubation in darkness, absorbance was assessed at 550 nm use a microplate reader (Thermo Fisher Scientific). Nitrite levels were determined using a standard curve generated from sodium nitrite.

### Western blot

The cells were lysed using 1 × RIPA lysis buffer (Serva, 39,244.01) containing 10% SDS (Applichem, A1112,0500), and 1% protease inhibitor (Applichem, A7779,0001), and their concentration was measured using the Pierce™ Rapid Gold BCA Protein Assay Kit (Thermos Fisher Scientific, A53225). Subsequently, 35 μg of the protein sample was loaded onto a 10% polyacrylamide gel, and the proteins were separated by sodium dodecyl sulfate–polyacrylamide gel electrophoresis (SDS–PAGE). For blotting, the proteins were transferred to 0.2 μm PVDF membranes (Biorad) using a Trans-Blot Turbo RTA Mini. The membranes were blocked with 5% skim milk for 1 h at room temperature and then incubated overnight at 4 °C with primary antibodies against NFκB (1:1000, Cell Signaling Technology, 8242), iNOS (1:1000, Cell Signaling Technology, 13,120), COX-2 (1:1000, Thermo Fisher Scientific, 35–8200), and GAPDH (1:10,000, Boster, M00227). The following day, the membranes were incubated with Goat Anti-Mouse IgG H&L (HRP) preadsorbed (1:1000, Abcam, ab97040) and goat anti-rabbit (1:1000, Boster, BA1054-0.5) secondary antibodies for 1 h at room temperature. The membranes were then incubated in WesternBright ECL-HRP substrate (Advansta, ADV-K-12045-C20) for 1–2 min and visualized using a C-Digit Blot Scanner (LICOR). Membrane stripping was conducted to reanalyze with different antibodies and to standardize target protein levels.

### Statistical analysis

All data are expressed as mean ± standard deviation (SD). Statistical analysis was performed using GraphPad Prism 9 software. One-way analysis of variance (ANOVA) was used to compare the differences among groups. Statistical significance relative to the control group is indicated by asterisks: *p < 0.05, **p < 0.01, ***p < 0.001, and ****p < 0.0001. Comparisons with the NI group are denoted by number signs: #*p* < 0.05, ##*p* < 0.01, ###*p* < 0.001, and ####*p* < 0.0001. Not significant differences were labeled as “ns.”

## Results

### Isolation and characterization of Exo and Que-Exo, and evaluation of Que-Exo cellular uptake

**Fig. 1:**
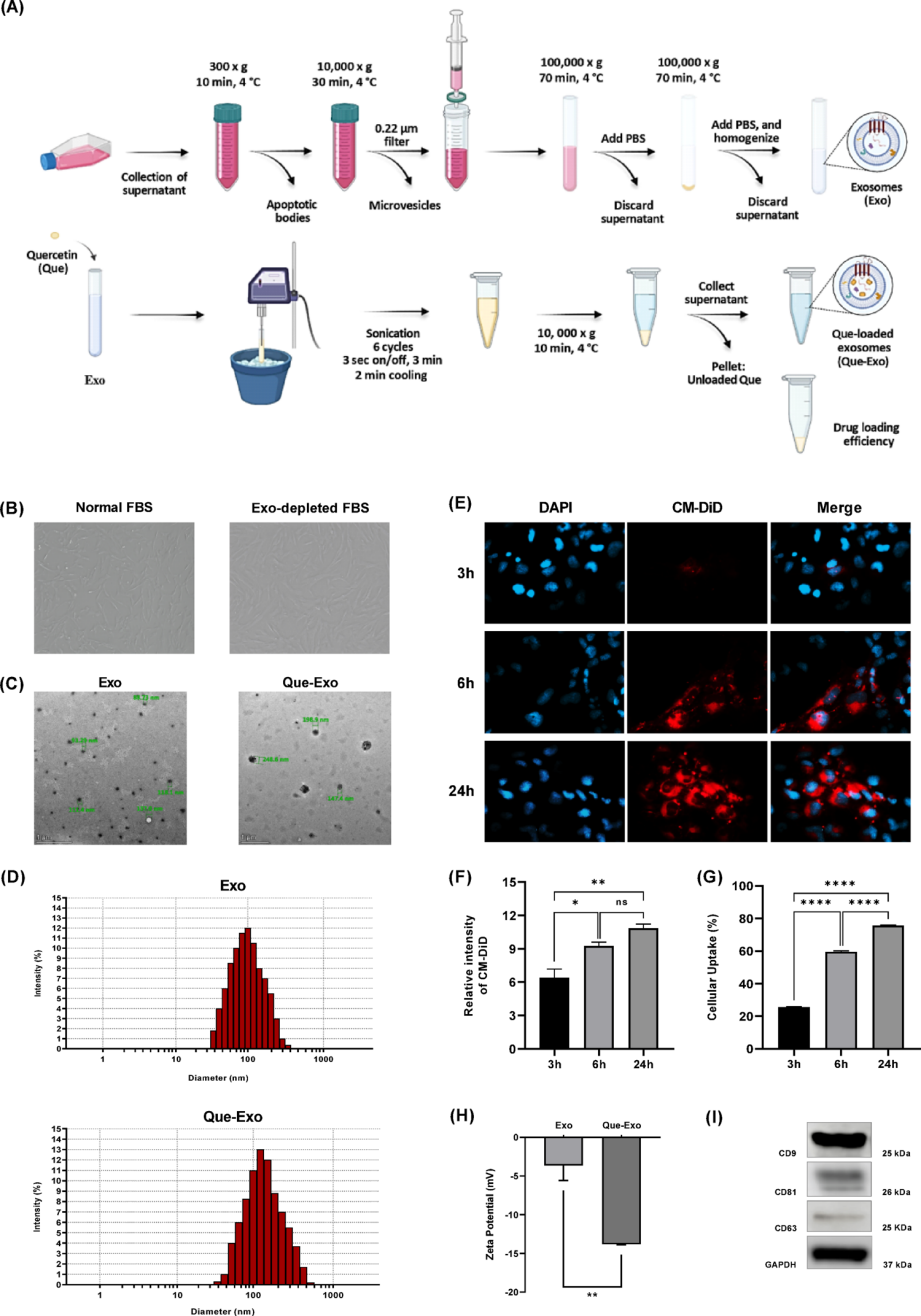
Isolation, characterization of Exo and Que-Exo, and evaluation of Que-Exo cellular uptake by HMC3 cells. **A** Schematic overview of Exo isolation and Que encapsulation. **B** Morphology of UC-MSCs cultured with normal FBS (passage 3) and Exo-depleted FBS (passage 5); images were captured at 5 × magnification. **C** TEM images of Exo and Que-Exo (scale bar: 1 μm). **D** Particle size distribution of Exo and Que-Exo measured by DLS. **E** Fluorescence microscopy of CM-DiD-labeled Que-Exo (red) internalized in DAPI-stained HMC3 cells (blue) at 3, 6, and 24 h (scale bar: 20 μm). **F** Fluorescence intensity was quantified as MFI and **G** cellular uptake was measured as the percentage of CM-DiD-positive cells expressed as a total cell population (n = 2). **H** Zeta potential analysis of Exo and Que-Exo (n = 3). **I** Detection of exosomal markers CD63, CD81, and CD9 by western blot (10 µg protein loading; n = 3). Data are presented as mean ± SD. Statistical significance is indicated as follows: ns, not significant; *p < 0.05; **p < 0.01; ****p < 0.0001

Exo were successfully isolated from the supernatant using ultracentrifugation and subsequently loaded with Que via sonication (Fig. 1A). Under normal cell culture conditions, UC-MSCs cultured with standard FBS exhibited typical fibroblastic morphology. However, when cultured with FBS depleted by ultrafiltration, the cells predominantly displayed an enlarged morphology, in contrast to the multipolar forms observed with regular FBS (Fig. 1B). TEM images revealed that both Exo and Que-Exo were similar in size, highly homogeneous, and exhibited spherical morphology (Fig. 1C). DLS analysis indicated mean diameter of 110 nm for Exo and 150 nm for Que-Exo (Fig. 1D). The size distribution of Exo was slightly altered following Que loading. In addition, a notable difference in particle concentrations was detected using DLS. Exo concentration was 4.2 × 10⁷ particles/mL, whereas Que-Exo concentration was 4.75 × 10^6^ particles/mL. The zeta potential, indicative of nanoparticle colloidal stability, was lower for the Que-Exo, with a measurement of -13.9 mV, compared to -3.63 mV for free Exo (Fig. 1H). Western blot analysis confirmed the successful isolation and characterization of Exo, with strong bands observed for CD9 and weaker but detectable signals for CD81 and CD63 (Fig. 1I).

Our data collectively confirmed the successful isolation of Exo from UC-MSCs. Following characterization, the time-dependent uptake of Que-Exo by HMC3 cells was investigated. The cells were incubated with CM-DiD-labeled Que-Exo and monitored at various time points using fluorescence microscopy (Fig. 1E-G). The CM-DiD dye selectively labeled the lipid membrane of Que-Exo, while DAPI staining was used to visualize cell nuclei. Initial Que-Exo internalization was observed at 3 h as a faint CM-DiD fluorescence signal within the perinuclear region, which intensified significantly by 24 h (Fig. 1E). MFI analysis showed a substantial increase from 3 to 24 h (Fig. 1F), accompanied by a rise in cellular uptake efficiency to approximately 75% at 24 h (Fig. 1G). Collectively, these findings demonstrate a time-dependent increase in microglial uptake of CM-DiD-labeled Que-Exo, becoming most pronounced at 24 h.

### In vitro release of Que-Exo

**Fig. 2:**
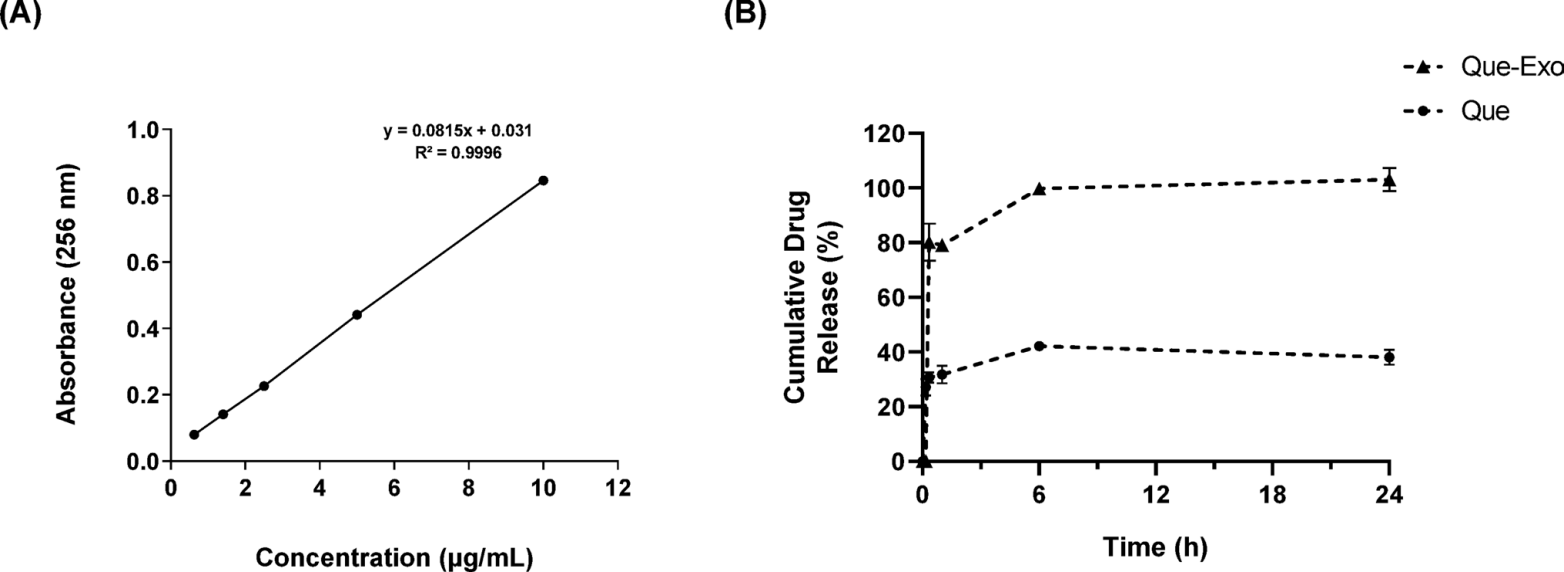
Stability and release studies of Que-Exo. **A** UV-Spectrophotometry calibration curve of Que at 256 nm. **B** Drug release profile of Que-Exo and free Que over 24 h. Data are presented as mean ± SD (n = 2)

The EE (%) of Que-Exo was quantitatively assessed using an indirect method, yielding a value of 46.26%. The successful encapsulation of nearly half of the initially introduced Que demonstrates efficient loading into the exosomal structure, likely facilitated by hydrophobic interactions between Que and the lipid bilayer of Exo. The concentration of Que was determined using a standard calibration curve at 256 nm (Fig. 2A). Comparative 24-h release profiles (Fig. 2B) revealed two phases: an initial burst release characterized by rapid drug discharge, and a controlled release marked by gradual, prolonged delivery over time. Release from Que-Exo began after 30 min, exhibiting a burst release followed by a controlled release over several hours. Que-Exo achieved approximately 100% release by 6 h and maintained this plateau for 24 h. In contrast, free Que showed a burst release within 10 min, reaching only 42.17% release at 6 h. The subsequent decline in free Que concentration is likely attributable to its inherent instability, poor solubility, or degradation in aqueous medium.

### Establishing an in vitro NI model and optimizing the concentrations of Exo and Que-Exo

**Fig. 3:**
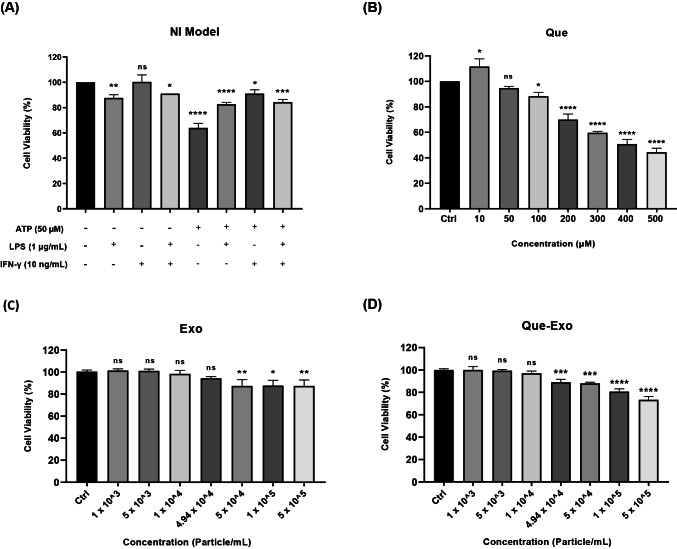
Evaluation of HMC3 cell viability under various treatment conditions. Cell viability was assessed via MTT assay following exposure to **A** inflammatory inducers (ATP, LPS, and IFN-γ), **B** Que, **C** Exo, and **D** Que-Exo. Data are presented as mean ± SD (n = 3). Statistical significance is indicated as follows: ns, not significant; *p < 0.05; **p < 0.01; ***p < 0.001; ****p < 0.0001 vs. control group

To identify a suitable neuroinflammatory inducer, HMC3 microglial cells were exposed to ATP, LPS, or IFN-γ **(**Fig. 3A**)**, and their effects on cell viability were assessed. ATP treatment markedly reduced cell viability, indicating pronounced cytotoxicity. In contrast, LPS caused a milder decrease in viability. Treatment with IFN-γ slightly enhanced cell viability, suggesting a potential stimulatory effect on microglial proliferation or survival under these conditions.

At a low concentration of 10 μM (Fig. 3B), Que significantly increased cell viability, indicating its cytoprotective effect. Nevertheless, with increasing doses, the compound began to exhibit cytotoxic properties, with significant toxicity observed at 100 μM. Consequently, higher concentrations were excluded from subsequent experiments, and 10 μM was selected as the working concentration. The impact of Exo and Que-Exo on HMC3 cell viability was assessed across particle concentrations ranging from 1 × 10^3^ to 5 × 10^5^ particles/mL, respectively (Fig. 3C and D). For Exo, concentrations between 1 × 10^3^ and 1 × 10^5^ particles/mL had no significant effect on HMC3 cell viability. However, a gradual decrease in cell viability was observed at 5 × 10^4^ and 5 × 10^5^ particles/mL. Similarly, Que-Exo concentrations between 1 × 10^3^ and 1 × 10^5^ particles/mL did not affect cell viability. Nevertheless, concentrations from 4.94 × 10^4^ to 5 × 10^5^ particles/mL showed dose-dependent toxicity.

A concentration of 4.94 × 10^4^ particles/mL was chosen as the working dose for both Exo and Que-Exo. This concentration was selected because, for Que-Exo, it corresponds to the particle number required to deliver 10 μM Que, a dosage previously shown to improve cell viability.

### Que-Exo alleviate LPS-induced inflammation in HMC3 cells

**Fig. 4:**
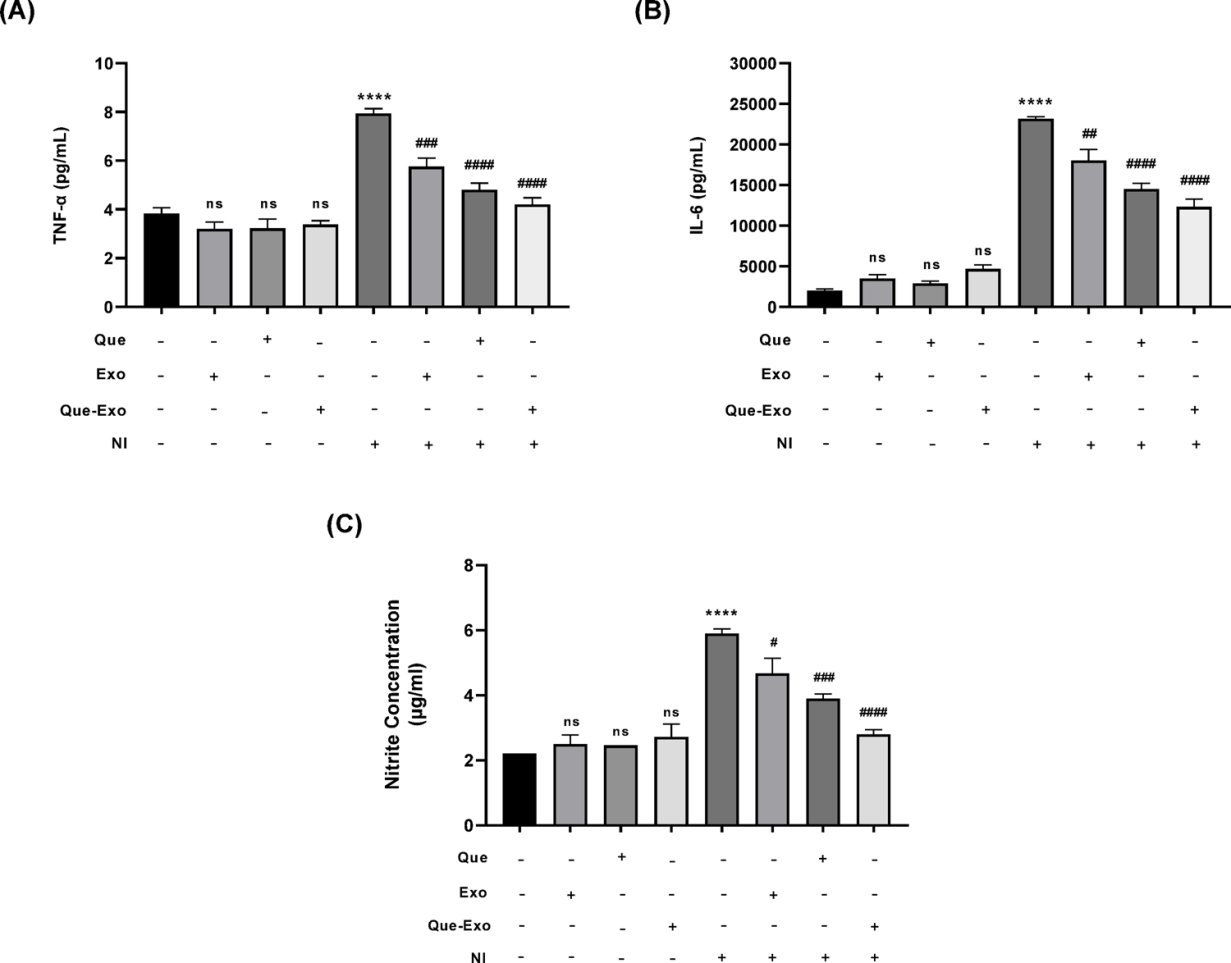
Que-Exo attenuate inflammatory mediators in HMC3 cells. Concentrations of **A** TNF-α and **B** IL-6 (n = 2), and **C** NO (n = 4) were measured using flow cytometry and Griess assay, respectively. Data are presented as mean ± SD. Statistical significance is indicated as follows: ns, not significant; ****p < 0.0001 vs. control group; #*p* < 0.05; ##*p* < 0.01; ###*p* < 0.001; ####*p* < 0.0001 vs. NI group

The expression levels of the pro-inflammatory cytokines TNF-α and IL-6 (Fig. 4A and B) were markedly elevated in the NI group following stimulation with LPS and IFN-γ. Specifically, TNF-α and IL-6 concentrations in the NI group were considerably higher than those in the unstimulated control group, indicating successful induction of an inflammatory response. In contrast, pretreatment with Exo or Que led to a modest reduction in TNF-α and IL-6 levels. Although both interventions appeared to exert a partial anti-inflammatory effect, the decreases were not statistically significant compared to the NI group. A pronounced and statistically significant decrease in TNF-α and IL-6 expression was observed in cells pretreated with Que-Exo. This substantial reduction highlights the enhanced therapeutic and synergistic efficacy of the combined formulation, suggesting that exosomal delivery of Que may improve its bioavailability and anti-inflammatory activity.

Furthermore, NO production was measured using the Griess reagent kit, which detects the primary reactive species synthesized by iNOS during inflammatory responses. NO levels were elevated in the NI group, confirming the successful induction of inflammation (Fig. 4C). Treatment with Exo, Que, and Que-Exo in the absence of inflammatory stimuli did not increase NO production compared to the control group. Pre-treatment with Exo and Que in the NI model slightly reduced NO concentrations compared to the NI group. Notably, pre-treatment with Que-Exo demonstrated a significant anti-inflammatory effect, markedly decreasing NO levels compared to the NI group.

### Que-Exo suppress the NF-κB mediated inflammation induced by LPS in HMC3 cells

**Fig. 5:**
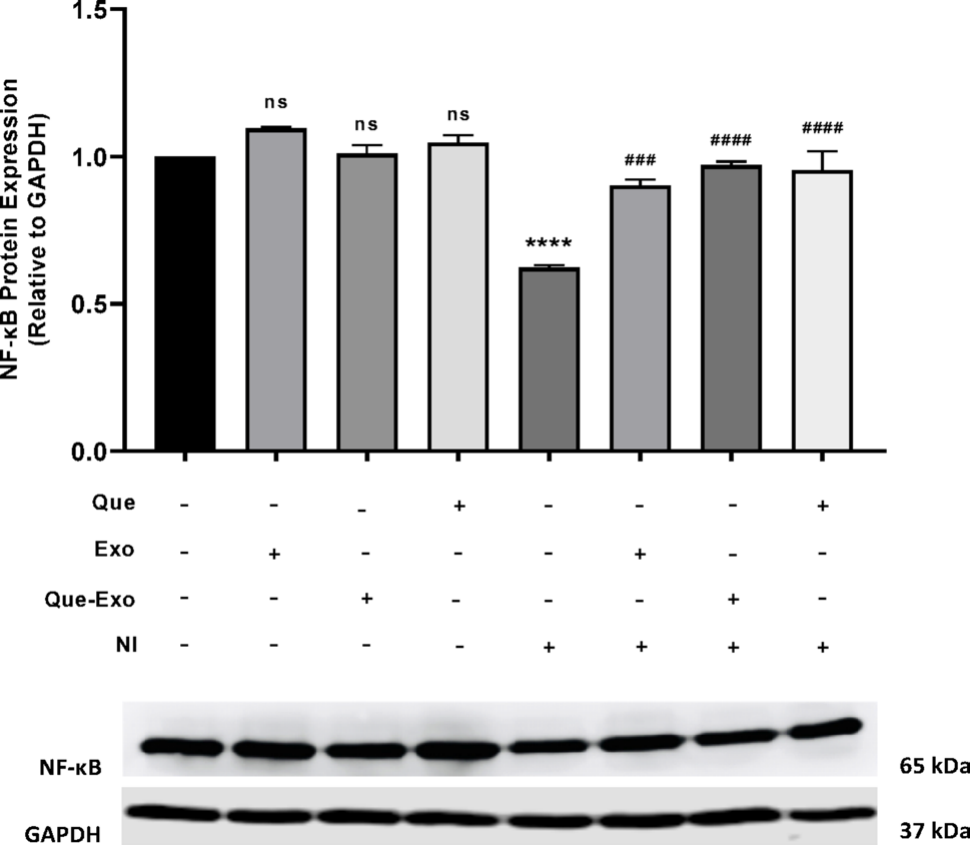
Que-Exo inhibit LPS-induced NF-κB activation in HMC3 microglial cells. NF-κB protein expression was determined by Western blot analysis and quantified by densitometry, with normalization to GAPDH (loading control). Data are presented as mean ± SD (n = 3). Statistical significance is indicated as follows: ns, not significant; ****p < 0.0001 vs. control group; ###*p* < 0.001; ####*p* < 0.0001 vs. NI group

According to the western blot results (Fig. 5), the NI group showed a reduction in cytoplasmic NF-κB expression compared to the control group, contrary to the expected increase associated with inflammatory activation. This suggests successful translocation of NF-κB from the cytoplasm to the nucleus. However, pretreatment with Exo, Que, and Que-Exo led to a slight increase in cytoplasmic NF-κB expression, indicating their potential to inhibit NF-κB nuclear translocation. By preventing this translocation, these treatments retained NF-κB in the cytoplasm, resulting in elevated cytoplasmic expression levels.

### Que-Exo inhibit pro-inflammatory enzymes iNOS and COX-2 in vitro

**Fig. 6:**
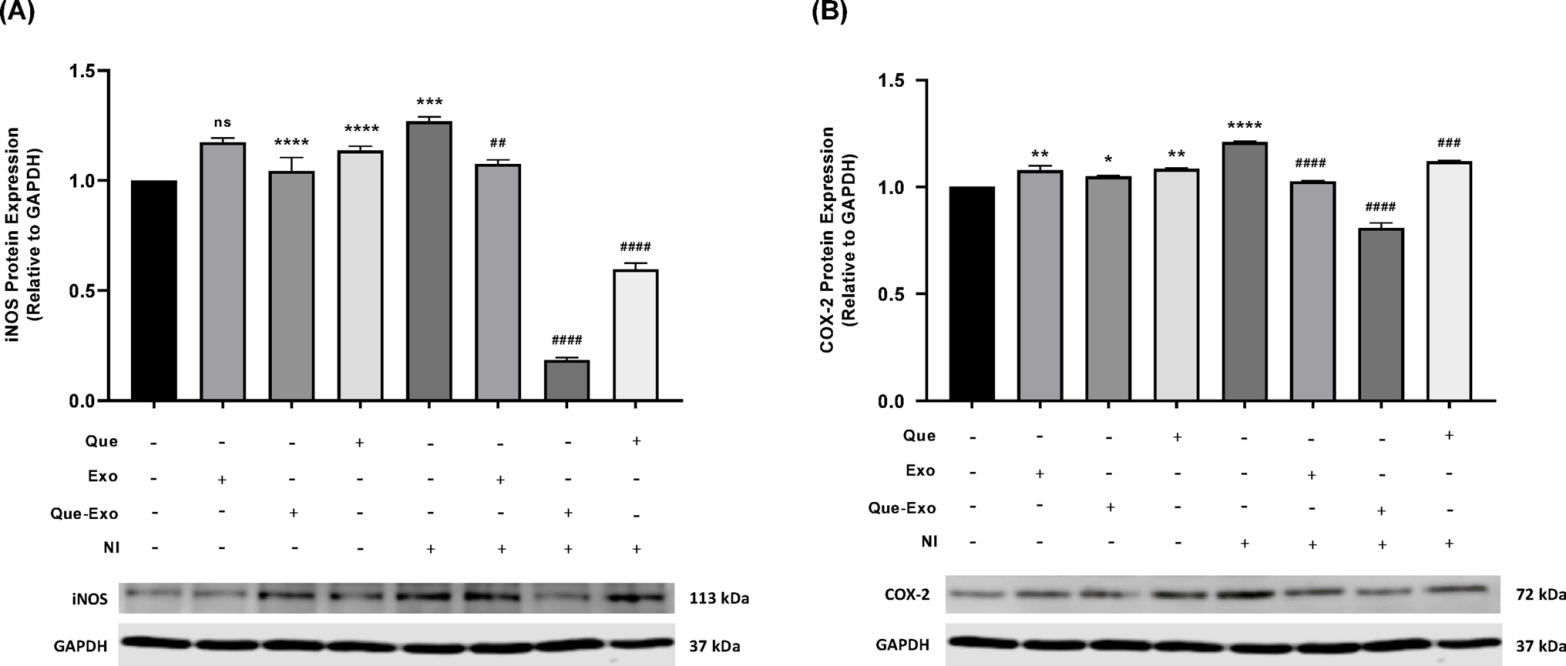
Que-Exo suppress pro-inflammatory protein expression in HMC3 cells. Protein levels of **A** iNOS and **B** COX-2 were assessed by Western blot analysis with densitometric values normalized to GAPDH (loading control). Data are presented as mean ± SD (n = 3). Statistical significance is indicated as follows: ns, not significant; *p < 0.05; **p < 0.01; ***p < 0.001; ****p < 0.0001 vs. control group; ##*p* < 0.01; ###*p* < 0.001; ####*p* < 0.0001 vs. NI group

The protein expression levels of iNOS and COX-2 were further analyzed using western blotting to evaluate their regulation under different treatment conditions. In the analysis of iNOS protein expression (Fig. 6A), a slight increase was observed in the NI group compared to the control group, indicating an elevation in the inflammatory response following the induction of the neuroinflammatory condition. Groups pretreated with Exo and Que showed a slight reduction in iNOS levels, suggesting a mild anti-inflammatory effect exerted by both agents individually. Notably, pretreatment with Que-Exo resulted in a significant decrease in iNOS expression compared to the NI group. Regarding COX-2 expression (Fig. 6B), a slight increase was detected in the NI group compared to the control group. Pretreatment with Exo and Que led to a mild decrease in COX-2 levels. Moreover, the group pretreated with Que-Exo demonstrated a significant decrease in COX-2 expression compared to the NI group. Although the reduction was not as pronounced as that observed for iNOS in the same group, it still suggests that Que-Exo can attenuate inflammatory signaling, due to a synergistic or enhanced anti-inflammatory effect resulting from the combination of Que and Exo-based delivery. This highlights a potential therapeutic advantage of Que-Exo in mitigating neuroinflammation.

## Discussion

Our study provides compelling evidence that Exo-mediated delivery of Que exerts significant anti-inflammatory effects in an in vitro model of neuroinflammation using the HMC3 human microglial cell line. Neuroinflammatory insults, commonly observed in NDs, are predominantly mediated by activated microglia (Han et al. 2021). Our inflammation model, previously optimized using ATP, LPS, and IFN-γ, successfully induced an activated microglial phenotype, as evidenced by elevated levels of proinflammatory cytokines and enzymes. Microglial activation results in the overproduction of neurotoxic substances, which intensifies neuronal injury through a mechanism known as reactive microgliosis (Kim et al. 2013).

Given that the properties of Exo can directly influence their anti-inflammatory potential, UC-MSCs are often preferred due to their higher proliferation rates and greater Exo yield compared to bone marrow- and adipose-derived MSCs (Haraszti et al. 2018). In addition, variability among UC-MSC donors can still affect Exo yield and composition. UC-MSCs obtained from donors of different ages secreted varying amounts of Exo under identical conditions, likely due to intrinsic differences in cellular activity and proliferation. Exo populations exhibit compositional heterogeneity, comprising distinct subpopulations that express canonical markers such as CD9, CD63, CD81, TSG101, and Alix, reflecting their biological complexity. Although variations in Exo yield exist, the underlying profile of these subpopulations remains remarkably consistent across individuals (Zhang et al. 2025). Exosomes from adipose-derived MSCs at early passages show enhanced proliferative and anti-apoptotic effects, whereas later passages may exhibit reduced therapeutic efficacy due to MSC senescence (Chen et al. 2022). Moreover, donor age and culture conditions, particularly passage number, may contribute to variability in exosome yield, as prolonged culture can impair the proliferation and differentiation capacities of bone marrow–derived MSCs (Zaim et al. 2012), potentially affecting exosome production and content. Therefore, similar effects are likely to occur in UC-MSCs.

Exosomes have been identified to exert promising effects in NDs (Quan et al. 2025). Notably, efficient delivery of Exo to the brain has enabled their selective targeting of dopaminergic neurons in Parkinson’s disease models (Peng et al. 2022). In this study, we employed ultracentrifugation to isolate Exo from MSCs. This method not only allows the efficient processing of large sample volumes but also minimizes contamination risks, making it both cost-effective and suitable for downstream applications (Ahmad et al. 2025). We successfully isolated and characterized sufficient quantities of Exo to support in vitro experiments. The high yield and quality of Exo produced using this method suggest strong potential for future therapeutic applications, particularly in the development of exosome-based treatment strategies. Furthermore, Exo have previously been evaluated in microglial cells under LPS-induced neuroinflammatory conditions, with various studies showing their potential to attenuate inflammatory responses (Thomi et al. 2019).

In this regard, Que, a natural flavonoid prevalent in fruits and vegetables, is recognized for its potent antioxidant and anti-inflammatory properties (Sanad et al. 2023), which may be further enhanced through exosomal delivery. However, factors such as poor aqueous solubility, low stability, and limited cellular uptake restrict its therapeutic application (Aghababaei and Hadidi 2023). Encapsulation of Que within Exo has been proposed as a strategy to enhance its bioavailability and intracellular delivery. In this study, sonication was employed as the loading method due to its high loading efficiency. This technique transiently disrupts the exosomal membrane, facilitating the incorporation of Que into the vesicles while preserving their structural integrity (Kim et al. 2016). This approach offers the potential to amplify the therapeutic benefits of Que by leveraging the biological compatibility and targeting capabilities of Exo. Following Que loading, we observed a slight increase in particle size and a shift in zeta potential toward more negative values, confirming successful entrapment. In addition, the particle concentration decreased approximately tenfold, a reduction likely attributed to the membrane disruption inherent to the sonication process.

Our drug release study demonstrated that the bioavailability of Que was significantly enhanced when delivered via Exo, compared to free Que, which exhibited lower bioavailability. Que-Exo achieved approximately 100% release by 6 h, likely due to the excellent dispersibility and compatibility of Exo in aqueous environments. On the contrary, Que showed low solubility in aqueous media, which may elucidate its failure to reach a 100% release rate. This limited solubility restricts its pharmacological efficacy. At neutral pH, Que remains stable for only about 4.5 h before undergoing hydrolysis and oxidation (Osojnik Črnivec et al. 2024). Consequently, the drug attained its saturation solubility within 6 h, restricting further release. No surfactants or organic solvents were used to enhance solubility; instead, a basic aqueous Que dispersion in PBS served as the control. These findings suggests that exosomal encapsulation of Que effectively improves its stability and enables controlled release, potentially overcoming its inherent solubility and absorption limitations. Nonetheless, the rapid release of approximately 80% from Que-Exo within 2 h likely reflects the in vitro sink conditions, which do not fully mimic the in vivo environment. In biological systems, factors such as plasma proteins, diffusion barriers, and osmolarity typically influence release kinetics. Therefore, these data do not confirm complete dissociation prior to cellular uptake, as some Que may remain associated with Exo or be co-internalized. Further studies under physiologically relevant conditions are essential to clarify these in vivo dynamics.

Fluorescence microscopy confirmed the efficient internalization of Que-Exo by HMC3 cells, demonstrated by an increase in intracellular fluorescence intensity over time. Successful cellular uptake is a critical prerequisite for the effective intracellular delivery of Que, enabling the nanocarrier to exert its therapeutic effects within the target cells. This uptake likely occurs via endocytosis, a common pathway for exosome internalization, which facilitates the release of Que into the cytoplasm, where it modulates signaling pathways. Ultimately, using an Exo-based delivery system improves the bioavailability of Que and overcomes the inherent challenges of poor solubility and rapid metabolism.

LPS is a bacterial endotoxin that activates NF-κB signaling by interacting with TLR4, and its exposure leads to microglial activation and promotes polarization toward a pro-inflammatory phenotype (Voss et al. 2016). In this state, microglia release elevated levels of pro-inflammatory cytokines and enzymes, contributing to neurodegeneration (Réus et al. 2015). In our study, we demonstrated that Que-Exo disrupted NF-κB signaling by preventing its translocation to the nucleus, a critical step in initiating inflammatory gene transcription. Instead, NF-κB was retained in the cytoplasm, as evidenced by increased cytoplasmic NF-κB expression following Que-Exo treatment. This cytoplasmic entrapment indicates that Que-Exo effectively block NF-κB nuclear activation, thereby attenuating downstream inflammatory cascades and reducing cytokine production. This finding was further supported by the notable downregulation of pro-inflammatory cytokines following Que-Exo administration. While pretreatment with either Exo or Que resulted in only a modest reduction in TNF-α and IL-6 expression, the combination exerted a markedly stronger inhibitory effect. This suggests a synergistic interaction, where exosomal encapsulation enhances cellular uptake and intracellular stability of Que, increasing its therapeutic efficacy.

Furthermore, Que-Exo significantly inhibited neuroinflammation at the transcriptional level. In our model, both iNOS and COX-2, pro-inflammatory enzymes transcriptionally regulated by NF-κB, were significantly reduced in the group treated with Que-Exo. This highlights that the anti-inflammatory effects of Que-Exo extend beyond cytokine suppression and involve deeper modulation of intracellular inflammatory pathways. Our experimental evidence indicates that the therapeutic efficacy of Que-Exo is primarily mediated through inhibition of the NF-κB signaling pathway. Although the involvement of Nrf2 activation and NLRP3 inflammasome suppression remains speculative in this study, future investigations are required to determine whether these mechanisms contribute to the anti-inflammatory potency of Que-Exo.

Our results highlighted that inflammatory stimulation increased NO concentrations, which serve as stable metabolites of NO. While individual treatments with either Exo or Que produced modest reductions in NO levels, Que-Exo pretreatment caused a significantly greater decrease. These findings underscore the critical role of targeting NO-mediated oxidative damage in microglia-driven neuroinflammation and support the therapeutic potential of Que-Exo as a combined antioxidant and anti-inflammatory agent. Despite evidence from an in vivo Alzheimer’s disease model supports the therapeutic potency of Que-Exo (Qi et al. 2020), further research is warranted to evaluate their effectiveness across other NDs.

Overall, our findings highlight the potential of Que-Exo as a nanotherapeutic agent for combating microglia-mediated neuroinflammation; however, several limitations should be addressed. Exo size was measured using DLS via multi-angle light scattering with the DynaPro NanoStar, leveraging its ability to provide high statistical sampling across a wide concentration range during sample preparation. Nevertheless, it should be noted that DLS can overestimate size in the presence of larger particles or aggregates. A notable limitation of this study is the relatively small sample size (n = 2) for cytokine analysis and uptake studies. Further validation with larger sample sizes is necessary to confirm these preliminary findings. Recognizing the inherent limitations of cell-based models, this study employed an in vitro model using HMC3 cells to establish proof of concept and identify key mechanistic pathways. Nonetheless, this model does not replicate the full complexity of neuroinflammation in vivo, including multicellular interactions and three-dimensional architecture. Future research employing in vivo models or physiologically relevant systems, such as spheroids or organoids, is crucial for validating Que-Exo mechanisms and assessing their translational potential.

## Conclusion

In conclusion, Que-Exo exhibited potent anti-inflammatory effects on microglial cells in an in vitro model of neuroinflammation. These effects are, at least in part, attributed to the inhibition of the NF-κB signaling pathway, which plays a central role in the transcription of inflammation-related genes. Que-Exo treatment effectively reduces the expression and secretion of pro-inflammatory cytokines and enzymes and suppresses NO production, all of which are key contributors to neuroinflammatory damage. By delivering Que in a stable and bioavailable form, Exo enhance their cellular uptake and anti-inflammatory potency. This targeted modulation of microglial activation offers a promising strategy for preventing inflammatory responses that contribute to neuronal dysfunction and degeneration in NDs. Thus, Que-Exo represent a promising nanotherapeutic agent for developing effective cell-free treatments aimed at mitigating neuroinflammation in the CNS.

## Data Availability

The datasets generated during and/or analysed during the current study are available from the corresponding author on reasonable request.
